# Anxiety, attitudes, and education about fertility among medical students in the United States

**DOI:** 10.1186/s12909-023-04075-w

**Published:** 2023-03-03

**Authors:** D. Grace Smith, Abigail Ross, Elena HogenEsch, Rachel Okine, Marissa L. Bonus, Eve C. Feinberg, Lia A. Bernardi

**Affiliations:** 1 Northwestern University, Feinberg School of Medicine, Chicago, IL USA; 2 Vanderbilt University, College of Arts and Science, Nashville, TN USA; 3 Northwestern University, McGaw Medical Center, Chicago, IL USA; 4 Northwestern University, Weinberg College of Arts and Sciences, Evanston, IL USA; 5 Department of Obstetrics and Gynecology, Division of Reproductive Endocrinology and Infertility, Northwestern University Feinberg School of Medicine, Chicago, IL USA

**Keywords:** Medical student, Family planning, Childbearing, Infertility

## Abstract

**Background:**

As delayed family building is common among physicians, the goal of this study was to evaluate childbearing plans, anxiety related to future fertility, and interest in fertility education among medical students.

**Methods:**

Using convenience and snowball sampling methods, an electronic REDCap survey was distributed via social media and group messaging applications to medical students enrolled in medical schools across the United States. Answers were collected, and analysis of the descriptive statistics was performed.

**Results:**

The survey was completed by 175 participants, 72% of which were female (assigned at birth). The mean (± SD) age of participants was 24.9 ± 1.9 years. Of all participants, 78.3% desire to have children and 65.1% of these individuals plan to delay childbearing. On average, the planned age of first pregnancy is 31.0 ± 2.3 years. “Lack of time” was the greatest influence on decision regarding timing of childbearing.

Of all respondents, 58.9% reported at least some anxiety related to future fertility. When females and males were compared, significantly more females (73.8%) versus males (20.4%) reported worrying about future fertility (*p* < 0.001). Participants reported that greater knowledge about infertility and potential treatments would help ease fertility related anxiety, and 66.9% of respondents showed interest in learning about how things such as age and lifestyle can impact fertility, preferably through medical curricula, videos, and podcasts.

**Conclusion:**

A majority of the medical students in this cohort intend to have children and most plan to delay childbearing. A large percentage of female medical students reported anxiety related to future fertility, but many students showed interest in receiving fertility education. This study highlights an opportunity for medical school educators to incorporate targeted fertility education into their curriculum with the intention of decreasing anxiety and improving future reproductive success.

**Supplementary Information:**

The online version contains supplementary material available at 10.1186/s12909-023-04075-w.

## Introduction

In recent years, the nation’s physician workforce has steadily increased to include more female physicians. The growth in female physicians is largely due to a noteworthy increase in female medical students matriculating into medical school [1]. These changing demographics have contributed to delayed family building becoming more commonplace in the medical field [2, 3]. Discussion of this phenomenon through both media sources and dialogue within the medical community has generated interest in family planning among medical trainees [4–8]. However, this increased interest does not directly correlate with increased factual knowledge of reproductive aging [8–12]. While the public has a broad understanding of the relationship between aging and fertility, knowledge regarding the individual factors that contribute to childbearing is limited even amongst well-educated individuals [13]. Misinformation about family planning is common, and there is often an overestimation of the cost and success rate of fertility treatment [5, 13]. While there have been significant calls to action regarding physician infertility [4, 5] and several studies have addressed medical student fertility knowledge [7, 8, 14], no studies have reported on the anxiety medical students may experience concerning their own reproductive health, nor has medical student interest in learning about fertility for personal reasons been assessed.

Delayed childbearing, increasing age at first birth, and prominence of Assisted Reproductive Technology (ART) use in family building are predicted to continue to grow, making accessibility to information about reproductive aging critical for future physicians who are likely to delay family building. Understanding childbearing plans, examining anxiety amongst, and identifying interest in fertility education in medical students is critical to developing appropriate education and promoting successful reproductive planning. In this study, we aimed to assess family building goals, baseline fertility knowledge, anxiety about fertility, and interest in fertility education in medical students who may delay childbearing due to career pursuits.

## Materials and methods

### Survey

An online survey containing questions relating to fertility and family building was created and administered to participants via Research Electronic Data Capture (REDCap). Using social media, group messaging applications, and online communications, this survey was released and completed anonymously. The survey was distributed from June through October 2021 using convenience and snowball sampling methods. Given this survey was distributed during the COVID-19 pandemic, only virtual methods of recruitment were employed. In the survey, participants were asked about their educational background, personal desires for children and plans to start a family, anxiety related to future reproduction, and interest in learning more about fertility. Participants also answered five questions designed to ascertained baseline knowledge regarding female age-related fertility decline. A sliding scale response from 0 (“strongly disagree”) to 100 (“strongly agree”) was used to assess participants’ reasons for delaying childbearing and attitudes towards anxiety related to reproduction and family building. Questions used in the survey can be found in the Supplemental Appendix.

### Participants

This survey was approved by the Institutional Review Board at Northwestern University. The survey was sent to young professional adults, ages 22 to 30, that were currently enrolled in or planned to attend graduate school. As such, this study was distributed across all regions of the continental United States. Participants who completed the survey were entered into a drawing to win a $50 gift card and five individuals were randomly selected to win.

### Statistical analysis

A sub cohort of participants that only included female and male medical students between the ages of 22 to 30 years old actively enrolled in a graduate medical school program was the target population for analysis. Exclusion criteria were participants graduated from medical school (resident physicians, fellows, and attending physicians) and medical students enrolled in international medical schools. Through REDCap, survey results were collected and evaluated using an analysis of descriptive statistics and two sample t-tests assuming unequal variance. Categorical variables have their proportions displayed. Likewise, continuous variables have their means with standard deviations displayed. Participant’s reasons for delaying childbearing and anxiety related to future fertility were measured on a sliding scale from 0 to 100. Mean scores from the sliding scale responses were calculated and reported. In addition, percentages of participants who reported a score greater than “50” on the sliding scale were also calculated; a response over “50” was deemed an endorsement of the survey question or statement. Every participant response was analyzed, and an additional statistical analysis was performed to compare the responses of males and females. A 95% confidence interval was utilized in the analysis to determine statistical significance with any *p*-value less than 0.05.

## Results

### Demographics

A total of 175 participants (126 females, 49 males, sex assigned at birth) with a mean age of 24.9 ± 1.9 years completed the survey (Table 1). Of all participants, 85.1% identified as heterosexual, and 13.1% identified as bisexual, gay, or lesbian. A majority of participants identified as Caucasian (65.7%), while 24.0% identified as Asian, and 5.7% identified as African American. Just over half of the respondents were single (61.7%), and no participants had children. Of the total participants, 96.6% were enrolled in allopathic medicine studies.

**Table 1:** Participant characteristics (*N* = 175)

Age, year, mean, + SD	24.9 + 1.9
**Sex at Birth**, n (%)	
Female	126 (72.0%)
Male	49 (28.0%)
**Gender Identity,** n (%)	
Female	122 (69.7%)
Male	49 (28.0%)
Non-Binary	3 (1.7%)
**Sexual Orientation**, n (%)	
Heterosexual/Straight	149 (85.1%)
Bisexual	20 (11.4%)
Gay/Lesbian	3 (1.7%)
Prefer not to answer	2 (1.1%)
**Relationship Status**, n (%)	
Single	108 (61.7%)
Married/Partnered	62 (35.4%)
Divorced	1 (0.6%)
Not listed	4 (2.3%)
**Racial Background**, n (%)	
Caucasian	115 (65.7%)
Asian	42 (24.0%)
Hispanic/Latinx	12 (6.9%)
African American	10 (5.7%)
Middle Eastern	7 (4.0%)
Multiracial other	2 (1.1%)
Other or prefer not to answer	1 (0.6%)
**Region**, n (%)	
Midwest	140 (80.0%)
West	17 (9.7%)
East	15 (8.6%)
South	0 (0.0%)
**Medical Degree**, n (%)	
MD	169 (96.6%)
DO	6 (3.4%)

### Childbearing plans

Of all participants, 78.3% reported a desire to have children. Of those who desired children, 65.1% planned on delaying childbearing, and there were no significant differences in plans to delay by gender (55.1% males, 69.0% females, *p* = 0.117). On average, participants stated that their ideal age at first pregnancy was 31.0 ± 2.3 years old; no participant reported planning to have their first child after the age of 38. A large majority (96.2% of participants) who answered this question desired to have children by age 35.

As shown in Table 2, when participants were asked about their reasons for delaying childbearing, “lack of time” was the greatest influence on participants’ plans (85.1%). There was no significant difference between male and female responses. “Lack of flexibility in schedule” was also a common reason to delay childbearing (81.1%) and there was also no significant difference by gender. However, significantly more females than males reported that “lack of support from colleagues”, “concerns about burdening colleagues with extra work”, and “reputational concerns” contributed to delaying childbearing (*p* = 0.004, *p* < 0.001, p < 0.001, respectively). The only factor that was significantly more likely to be reported by males than females as a reason to delay childbearing was “financial strain” (65.3% versus 54.0% for males and females, respectively, *p* = 0.03). When comparing responses from Caucasian participants versus participants who did not identify as Caucasian, there were no significant differences across reasons for delaying childbearing, except for non-Caucasian participants reported that lack of romantic partner influences their decisions about the timing of childbearing significantly more than Caucasian participants (*P* < .05).

**Table 2:** Reasons for delaying childbearing

	Females		Males		***P*** value^**c**^
	Mean^**a**^ Score	Percent^**b**^	Mean^**a**^ Score	Percent^**b**^	
Does lack of time influence your decisions about the timing of childbearing?	77.5	88.9%	74.0	75.5%	NS
Does lack of flexibility in schedule influence your decisions about the timing of childbearing?	78.4	84.9%	75.6	71.4%	NS
Does financial strain influence your decisions about the timing of childbearing?	58.8	54.0%	69.5	65.3%	0.032
Does lack of romantic partner influence your decisions about the timing of childbearing?	42.0	36.5%	49.5	42.9%	NS
Does lack of support from colleague influence your decisions about the timing of childbearing?	36.2	31.0%	23.7	10.2%	0.004
Does concern about burdening colleagues with extra work influence your decisions about the timing of childbearing?	37.2	31.0%	21.0	6.1%	<.001
Do reputational concerns (e.g., being perceived as less committed to career) influence your decisions about the timing of childbearing?	49.2	51.6%	16.8	6.1%	<.001
Does being not ready for children influence your decisions about the timing of childbearing?	68.1	75.4%	75.8	73.5%	NS

(NS = *P* value not statistically significant)

^a^Mean score on a sliding scale where response could range from 0 (“Not at all”) to 100 (“Extremely”)

^b^Percentage of participants who reported a response greater than “50” on the scale which was deemed an endorsement of the survey question

^c^*P* value refers to mean score

### Fertility knowledge

While a large majority of participants (74.9%) correctly identified when a woman’s ability to conceive declines most precipitously, 37.1% of participants overestimated the chance of pregnancy per cycle in women ages 30 to 34 and 19.4% of participants overestimated the likelihood of pregnancy per cycle in women ages 40 to 43 (in the setting of properly timed intercourse and normal semen parameters). Medical students reported largely relying on their formal education to learn about the impact of age on fertility (66.3%), while 45.7% reported relying on the experiences of family and friends as resources.

### Concerns about future fertility and interest in fertility education

Of all respondents, 58.9% reported at least some anxiety related to future fertility. Overall, female participants reported significantly more worry over their future fertility (Table 3). Furthermore, females reported significantly greater degrees of anxiety related to future fertility because of career aspirations and related to the cost of infertility treatments than males. In general, female and male participants reported similar anxiety levels regarding partner status. There were no significant differences between Caucasian participants and participants who did not identify as Caucasian when responding to questions about anxiety related to future fertility. Participants reported that greater knowledge of infertility and potential treatments would help ease fertility related anxiety (42.8%) and 66.9% showed interest in learning about how things such as age and lifestyle can impact future fertility, preferably through medical school courses, educational videos, and podcasts.

**Table 3:** Anxiety related to future fertility

	Females		Males		***P*** value^**c**^
	Mean^**a**^ Score	Percent^**b**^	Mean^**a**^ Score	Percent^**b**^	
I worry about my future fertility.	65.1	73.8%	26.3	20.4%	< 0.001
I have anxiety related to my future fertility as a result of my career aspirations.	61.6	68.3%	22.0	14.3%	< 0.001
I have anxiety related to my future fertility related to my partner status.	35.9	32.5%	32.7	32.7%	NS
I have anxiety related to my future fertility related to the cost of infertility treatments.	39.2	31.7%	21.3	12.2%	< 0.001

(NS = *P* value not statistically significant)

^a^Mean score on a sliding scale where response could range from 0 (“strongly disagree”) to 100 (“strongly agree”)

^b^Percentage of participants who reported a response greater than “50” on the scale which was deemed an endorsement of the survey statement

^c^*P* value refers to mean score

## Discussion

While delayed childbearing may contribute to higher infertility rates among physicians, Stentz et al. demonstrated higher rates of infertility among physicians in aged-matched cohorts [5]. Previous data demonstrate that U.S. female physicians have their first child at an average age of 30.4 years, which is 6 years later than the general population [5, 15]. Similarly, our study found that while most medical students desired children, most planned to delay family building with an ideal age for first pregnancy of 31 years. Student doctors’ plans to delay childbearing are multifactorial, but our findings suggest female medical students feel pressure to delay pregnancy in part because of lack of support from colleagues, concern about burdening colleagues with extra work, and reputational concerns. More specifically, although both sexes plan to delay family building, female medical students report concern over being perceived as less committed to their career as a significant factor in their decision. Within the United States, the time-intensive nature of medical training is compounded by a lack of support for expectant mothers and inadequate maternity leave [16, 17]. It is likely that even during medical school, female students perceive this discrepancy in societal and institutional support for women in medicine who desire children. This perception is then reflected in their decision to delay childbearing and thus remain “committed” to their careers.

A large cohort study of female physicians found that 1 in 4 had been diagnosed with infertility [5]. Almost half of participants expressed some degree of regret regarding their approach to conception and childbearing, including not attempting conception earlier in their training. Our study confirms intentions to delay childbearing amongst medical students and identified significantly higher rates of anxiety regarding future fertility among female medical students. These findings are consistent with previous studies demonstrating that women, in general, report higher levels of infertility distress compared to men [18]. However, ours is the first study to report on fertility related anxiety of future physicians and to directly compare males with females. Female students’ anxiety largely centers around potential future infertility as a result of career aspirations, as well as the cost of infertility treatments. Therefore, the importance of addressing these findings among medical students is twofold; first, to decrease current stress levels of medical students and second, to promote long-term emotional, physical, and reproductive health amongst female physicians. Additionally, comprehensive insurance coverage for infertility should be accessible to add and finances should not be a barrier to family building.

Previous studies have shown that women who pursue graduate school are more likely to delay childbearing but have limited fertility and fecundity knowledge [8–11]. Similarly, we found that fertility knowledge was limited among both male and female medical students. Promisingly, our survey identified a strong desire to learn more about reproductive health and fertility within this cohort, specifically through the medical school curriculum. These findings highlight an important opportunity to incorporate fertility education into medical school curricula. The development of a pilot program for medical students that centers around the impact of age and lifestyle on fertility is a potential next step to empowering medical students with fertility knowledge. The implementation of such a curriculum would not only better serve patients but would also equip student physicians with the knowledge to secure their own reproductive success, potentially decreasing anxiety by improving knowledge [4, 14].

Strengths of this study include the detailed nature of the survey questions that help elucidate specifics of medical student plans to delay childbearing and their related anxieties. Furthermore, this study included a moderate cohort of male medical students which allowed for a more robust interpretation of the differences in fertility knowledge and stressors between male and female future physicians. This study is limited by potential selection bias, as those who chose to participate may have been more interested in, or anxious regarding their own fertility, thus potentially overestimating medical student interest in infertility education and knowledge of fertility. However, this risk is mitigated by the likelihood that students who are interested in learning may not have filled out the survey, and conversely students uninterested in learning about fertility may have filled out the survey strictly due to monetary motivations. The number of participants in this study was modest and heavily sampled from midwestern medical schools. Finally, although the survey was created by two Reproductive Endocrinologists and a portion of the questions were extracted from the Cardiff Fertility Knowledge Scale, every question was not externally validated. Further large-scale studies are needed to better reveal national attitudes towards fertility among medical students and interest in implementing fertility education into the medical school curriculum.

## Conclusions

The majority medical students sampled intend to have children, and the majority plan to delay childbearing. A large percentage of female medical students reported anxiety related to future fertility. Although fertility knowledge was limited in this cohort, many students showed interest in receiving fertility education. This study highlights a practical opportunity for medical school educators to actively incorporate target fertility education into their curriculum, with the intention of decreasing anxiety and improving future reproductive success.

## Supplementary Information

**Additional file 1.**

## Data Availability

The data that support the findings of this study are available from the corresponding author, D.G.S., upon reasonable request.
