# Supplementary material for: Anxiety, attitudes, and education about fertility among medical students in the United States
Source: BMC Med Educ. 2023 Mar 3;23:147. doi: 10.1186/s12909-023-04075-w (PMC9984243; doi:10.1186/s12909-023-04075-w)
Supplement: Supplementary file 1 — Additional file 1. [file 12909_2023_4075_MOESM1_ESM.pdf]

The goal of this survey is to assess fertility knowledge with the intention of determining how to best educate young professionals on fertility. Please complete this survey WITHOUT using external websites or information.

1. Are you currently completing this survey in the United States?
    a. Yes
    b. No – end survey
2. What is your age?
    a. Under 22 – end survey
    b. Over 30 – end survey
3. What state do you live in currently?
4. Have you received your undergraduate degree?
    a. Yes
    b. No – end survey
5. Are you a current graduate student?
    a. Yes
    b. No
6. If no, what is your current occupation?
    a. Free Text
7. Do you plan on attending graduate school within the next 5 years?
    a. Yes
    b. No
8. If yes, what degree will you pursue?
    a. MD
    b. DO
    c. PhD
    d. JD
    e. MBA
    f. MPH
    g. MS
    h. Other
9. Have you completed graduate school?
10. What year did you complete your graduate studies?
    a. Free Text
11. From question 6 [YES]: What year will you complete your graduate studies?
    a. 2021
    b. 2022
    c. 2023
    d. 2024
    e. Other
12. Professional degree achieved or currently pursuing (mark all that apply)
    a. MD
    b. DO
    c. PhD
    d. JD
    e. MBA
    f. MPH
    g. MS
    h. Other
13. If you have received your MD/DO, are you still in training?
    a. Yes
    b. No
14. If no, are you an attending?
    a. Yes

b. No
15. If yes, are you a resident or fellow?
    a. Resident
    b. Fellow
16. What is your postgraduate year?
17. MD/DO – specialty
    Anesthesiology
    Dermatology
    Emergency Medicine
    Family Medicine
    General Surgery
    Internal Medicine
    Obstetrics and Gynecology
    Ophthalmology
    Orthopedic Surgery
    Otolaryngology
    Pathology
    Pediatrics
    Physical Medicine and Rehabilitation
    Psychiatry
    Radiology – Diagnostic
    Radiology – Interventional
    Urological Surgery
    Other

DEMOGRAPHICS

What is your current gender identity?
Female

Male
Non-binary
Genderfluid
Not listed (please specify) ____
Prefer not to answer

What was your assigned sex at birth?
Male
Female

What is your sexual orientation?
Heterosexual (or straight)
Gay or lesbian
Bisexual
Not listed (please specify) ____
Prefer not to answer

With which race/ethnicity do you identify? (Please check all that apply)
- White
- Black or African American
- Asian
- American Indian or Alaska Native
- Hispanic or Latino
- Native Hawaiian or Other Pacific Islander
- Middle Eastern or North African
- Multiracial
- Not Listed (please specify) ________________
- Prefer not to answer

Which of these categories best describes your total combined household income for the past 12 months?
< $99K
$100K - $250K
$250K - $500K
> $500K
Don't know
Prefer not to answer

What is your current relationship status?
- Married/Partnered
- Single
- Divorced/Separated
- Widowed
- Not Listed (please specify)

How many hours a week do you work?
- 1 to 19 hours

20 to 39 hours
40 to 59 hours
> 60 hours/week
Not employed
Furloughed
Retired
Not Listed (please specify)

Do you have children (e.g., biological, adopted, step, or foster children)?
Yes
No
Prefer not to answer

Display This Question:
If Do you have children (e.g., biological, adopted, step, or foster children)? = Yes

How many biological children do you have?
0
1
2
3
4
5+

Display This Question:
If Do you have children (e.g., biological, adopted, step, or foster children)? = Yes

How many adopted, step, or foster children do you have?
0
1
2
3
4
5+

Display This Question:
If Do you have children (e.g., biological, adopted, step, or foster children)? = Yes

Do you intend to have additional children in the future? (e.g., biological, adopted, step, or foster)?
Yes
No
Undecided
Prefer not to answer

Display This Question:
If Do you intend to have additional children in the future? (e.g., biological, adopted, step, or fos… = Yes

How many additional biological children do you plan to have in the future?

0
1
2
3
4
5+

Display This Question:
If Do you have children (e.g., biological, adopted, step, or foster children)? = No

Do you intend to have children in the future? (e.g., biological, adopted, step, or foster)?
Yes
No
Undecided
Prefer not to answer

Display This Question:
If Do you intend to have children in the future? (e.g., biological, adopted, step, or fos… = Yes

How many biological children do you plan to have in the future?
0
1
2
3
4
5+

Display This Question:
If What was your assigned sex at birth = Female

How many times have you been pregnant (if any)?
0
1
2
3
4
5+

Display This Question:
How many times have you been pregnant (if any) > = 1

What were the outcome(s) of the pregnancy/pregnancies? (Please select all that apply).
Live born
Miscarriage
Termination
Not Listed

Display This Question:
How many times have you been pregnant (if any) > = 1

Did you delay having children because of your training or career? (Please select all that apply).
Yes, I have delayed in the past
Yes, I am currently still delaying
No
Other (please specify) ____________
Prefer not to answer

Display This Question:
If Did you delay having children because of your medical training or career? = Yes, I delayed in the past OR
If Did you delay having children because of your medical training or career? = Yes, I am currently still delaying

How long did you delay (or plan to delay) having children?
0 to 3 years
3 to 5 years
5+ years

Display these Questions:
If Do you have children (e.g., biological, adopted, step, or foster children)?
No, OR
If Do you intend to have children in the future (e.g., biological, adopted, step, or foster children)?
Yes OR Undecided

Do you plan to delay childbearing?
Yes
No

What age do you plan to conceive?
Free text

Do the following factors influence your decisions about the timing of childbearing?
(Sliding scale from 0 to 100 used on actual survey)

	Not at all	A little bit	Moderately	Very Much	Extremely
Lack of time	○	○	○	○	○
Lack of flexibility in schedule	○	○	○	○	○
Financial strain	○	○	○	○	○
Lack of romantic partner	○	○	○	○	○
Lack of support from colleagues	○	○	○	○	○
Concern about burdening colleagues with extra work	○	○	○	○	○
Reputational concerns (e.g., being perceived as less committed to career	○	○	○	○	○
Not ready for children	○	○	○	○	○

ANXIETY

Indicate the degree to which you agree or disagree (strongly disagree, disagree, neutral, agree, strongly agree) (Sliding scale from 0 to 100 used on actual survey)

18. I worry about my future fertility.
19. I have anxiety related to my future fertility as a result of my career aspirations.
20. I have anxiety related to my future fertility related to my partner status
21. I have anxiety related to my future fertility related to the cost of infertility treatments.
22. Which of the following would decrease your anxiety related to your future fertility?
    - Greater knowledge of infertility and potential treatments
    - Consultation with a fertility physician
    - Egg freezing
    - Support from partner
    - Support from friends/family members
    - Other

Fertility Knowledge Interest

1. I am interested in learning about how things such as age and lifestyle can impact my future fertility
    a. Yes
    b. No
2. [If yes to question 1] What would be your preferred method learning about fertility?
    - Educational video
    - Podcast
    - Interactive in person informational session
    - Interactive online informational session
    - Online infographics/brochures
    - Articles
    - As part of a medical school course
    - Other (please list)
3. Would you be interested in learning about resources for LGBTQ+ couples or how to pursue pregnancy or family building as a single parent? (surrogacy, sperm donors, egg donors, etc.)
    a. Yes
    b. No

Display This Question Block:
If What was your assigned sex at birth = Female

Have you ever considered egg/embryo freezing for fertility preservation?
- Yes
- No
- Prefer not to answer

Display This Question:
If Have you ever considered egg/embryo freezing for fertility preservation? = Yes

Did you seek consultation for egg/embryo freezing at a fertility center?
- Yes
- No
- Other (please specify)
- Prefer not to answer

Display This Question:
If Have you ever considered egg/embryo freezing for fertility preservation? = Yes

Did you freeze your eggs/embryos?
- Yes
- No
- Other (please specify)
- Prefer not to answer

Display This Question:
Did you freeze your eggs/embryos = Yes

At what age did you freeze your eggs/embryos?

____

Display This Question:
Did you freeze your eggs/embryos = No
Or Did you freeze your eggs/embyros = Other

Do you plan to freeze your eggs in the future?
- Yes
- No
- Don't know
- Other (please specify) ____
- Prefer not to answer

Display This Question:
Do you plan to freeze your eggs in the future = Yes

At what age do you plan to freeze your eggs/embryos?

____

Is egg/embryo freezing covered by your health insurance?
- Yes
- No
- Don't know

Are you aware of the approximate out-of-pocket cost that is associated with freezing your eggs/embryos?
1. Yes
2. No

If you learned that egg/embryo freezing was covered by your health plan, would you (or your partner) use it?

3. Yes
4. No
5. Not sure

Display This Question Block:
If What was your assigned sex at birth = Female

Have you ever experienced infertility?

Yes
No
Have not tried to conceive
Not applicable
Prefer not to answer

Display This Question:
If Have you ever experienced infertility? = Yes

Have you ever used in vitro fertilization (IVF) for conception?

Yes
No
Not applicable
Prefer not to answer

Display This Question:
If Have you ever used in vitro fertilization (IVF) for conception? = Yes

At what age did you undergo in vitro fertilization (IVF)?

Northwestern Medicine

Thank you for sharing your personal experiences. We are also interested in assessing knowledge of fertility and fertility preservation and the sources that individuals rely on to learn this knowledge. We hope this information may help inform our understanding of how to better educate young professionals on the role of age in fertility and fertility preservation.

Over which age range does a woman's ability to get pregnant decline most precipitously?
- 25 to 29 years old
- 30 to 34 years old
- Over 35 years old

What is the likelihood of pregnancy in a month with properly timed intercourse with normal semen analysis if a female is:

	<5%	10%	15%	25%	40%	60%	80%
30 to 34 years old	○	○	○	○	○	○	○
35 to 39 years old	○	○	○	○	○	○	○
40 to 43 years old	○	○	○	○	○	○	○
43 to 45 years old	○	○	○	○	○	○	○

Which of these have you relied on as sources to learn about the role of age in fertility?

	Yes	No
Formal education	○	○
Your own healthcare provider (e.g., OBGYN, PCP)	○	○
Your own personal experiences	○	○
Experiences of family and friends	○	○
Experiences of patients	○	○
Experiences of female physician colleagues	○	○
Internet	○	○
News outlets	○	○
Television/film	○	○
Individual posts from social media (e.g., Instagram, Twitter, Facebook)	○	○
Educational content from social media (e.g., Instagram, Twitter, Facebook)	○	○

Which of these have you relied on as sources to learn about elective egg freezing?

	Yes	No
Formal education	○	○
Your own healthcare provider (e.g., OBGYN, PCP)	○	○
Your own personal experiences	○	○
Experiences of family and friends	○	○
Experiences of patients	○	○
Experiences of female physician colleagues	○	○
Internet	○	○

News outlets	○	○
Television/film	○	○
Individual posts from social media (e.g., Instagram, Twitter, Facebook)	○	○
Educational content from social media (e.g., Instagram, Twitter, Facebook)	○	○

Email Instrument Survey

Please enter your email address if you wish to be entered into a raffle drawing (optional)

END SURVEY
